# Effects of Wing Kinematics on Aerodynamics Performance for a Pigeon-Inspired Flapping Wing

**DOI:** 10.3390/biomimetics10050328

**Published:** 2025-05-17

**Authors:** Tao Wu, Kai Wang, Qiang Jia, Jie Ding

**Affiliations:** 1 Northwest Institute of Mechanical and Electrical Engineering, Xianyang 712099, China; gdwutao@163.com (T.W.);; 2 Norinco Group Air Ammunition Research Institute Co., Ltd., Harbin 150030, China; dj986992@126.com

**Keywords:** flapping wing, unsteady flow, aerodynamic performance, twisting motion, sweeping motion

## Abstract

The wing kinematics of birds plays a significant role in their excellent unsteady aerodynamic performance. However, most studies investigate the influence of different kinematic parameters of flapping wings on their aerodynamic performance based on simple harmonic motions, which neglect the aerodynamic effects of the real flapping motion. The purpose of this article was to study the effects of wing kinematics on aerodynamic performance for a pigeon-inspired flapping wing. In this article, the dynamic geometric shape of a flapping wing was reconstructed based on data of the pigeon wing profile. The 3D wingbeat kinematics of a flying pigeon was extracted from the motion trajectories of the wingtip and the wrist during cruise flight. Then, we used a hybrid RANS/LES method to study the effects of wing kinematics on the aerodynamic performance and flow patterns of the pigeon-inspired flapping wing. First, we investigated the effects of dynamic spanwise twisting on the lift and thrust performance of the flapping wing. Numerical results show that the twisting motion weakens the leading-edge vortex (LEV) on the upper surface of the wing during the downstroke by reducing the effective angle of attack, thereby significantly reducing the time-averaged lift and power consumption. Then, we further studied the effects of the 3D sweeping motion on the aerodynamic performance of the flapping wing. Backward sweeping reduces the wing area and weakens the LEV on the lower surface of the wing, which increases the lift and reduces the aerodynamic power consumption significantly during the upstroke, leading to a high lift efficiency. These conclusions are significant for improving the aerodynamic performance of bionic flapping-wing micro air vehicles.

## 1. Introduction

The flapping kinematics of bird wings is of great significance for their excellent unsteady aerodynamics. Studying the mechanics behind the flapping motion of bird wings can not only reveal the mystery of natural biological flight, but also provide important theoretical guidance for the design and performance improvement of bionic flapping-wing micro air vehicles.

To reveal the aerodynamic mechanism of bird flight, careful observation and study of their wing morphology and motion are indispensable. In the early stage, the geometric shape data of different birds’ wings, including wing plane shape, wing area, and wing profile, were obtained through the measurement of bird wing specimens [1,2,3,4,5]. At this stage, the static morphological data were analysed mainly by means of constant or quasi-constant aerodynamics to study the steady gliding flight performance of birds. Due to the differences in geometric shape between the wing specimens and the real birds’ wings and the lack of observation of real wing kinematics, people did not have the conditions to study the aerodynamic mechanism of flapping flight in depth at the early stage. With the advancement of technology, people began to measure the shape and motion of birds’ wings during free flight through high-speed cameras, using stereo photography [6,7,8,9,10]. The dynamic shape changes and the acquisition of kinematic patterns gradually made it possible to study the mechanism of high thrust and large lift during flapping flight.

Researchers have observed that the typical flapping motion of bird wings consists of waving, twisting, sweeping, and folding [11,12,13,14]. Earlier, researchers revealed the mechanism of thrust generation from flapping wings by simplifying the waving motion of bird wings into the plunging motion of a 2D wing profile [15,16] and further investigated the effect of motion parameters (e.g., flapping frequency and St) on the magnitude of thrust generation from a plunging and pitching wing [17]. By reducing 3D wing flapping and twisting motions to 2D wing plunging and pitching motions, it was found that their coupling could lead to a significant improvement in thrust performance [18], and the optimal propulsive efficiency can be obtained when the phase difference between plunging and pitching is about 90° [19]. Research on the flapping motion of a 3D seagull-like wing has revealed that the dynamic twisting motion significantly enhances the thrust and propulsion efficiency of the flapping wing [20]. Further studies have shown that the forward sweeping motion can also increase the lift during the downstroke of a slow flight, and the sweeping motion pattern is automatically adjusted to change the aerodynamic effect as the flight speed changes [21]. The folding motion of the bird’s wings leads to dynamic changes in the span length, and it was found that the folding motion increases the instantaneous and average lift coefficients of the wings through a simplified model of variable span flapping [22]. Further studies have shown that the folding motion of wings can also increase the efficiency of lift generation and reduce energy consumption [23,24].

Although some conclusions on the aerodynamic mechanism of the flapping motion mentioned above have been achieved, most studies are based on simplified models or simplified flapping kinematics, with only one or two motions superimposed on the flapping wing, which does not fully reveal the aerodynamic mechanism of flapping flight. Firstly, simple 2D models are widely used in research, which ignores the 3D flow effects of flapping wings and cannot truly reflect the effects of the flapping motion on the leading-edge and wingtip vortices, while the accurate capture of complex vortex systems is very important for revealing the mechanism of high lift and large thrust generation. In addition, most studies only superimpose one motion on the flapping motion to study its aerodynamic effects, which ignores the coupling effect of multi-degree kinematics, while bird wings often realize flapping, twisting, sweeping, and folding at the same time to exert their aerodynamic advantages through coupled motion.

To address these issues, further investigation on a model which is closer to the real shape and kinematics of a bird wing was performed in this article. First, the dynamic geometry of the flapping wing was reconstructed based on the pigeon profile and planar shape parameters, and the kinematic parameters of the 3D pigeon wing were extracted from the trajectories of the wingtips and wrists during the cruising flight of a flying pigeon. Then, a hybrid RANS/LES method was used to study the effects of the wing twisting motion and the sweeping motion on the aerodynamic performance and flow pattern of the flapping wing.

This article continues in Section 2 to describe the kinematic models and the numerical simulation method used in this work. In Section 3, the effects of different wing kinematics on aerodynamic performance are investigated. In Section 4, the conclusions are presented.

## 2. Methodology

### 2.1. Geometry and Kinematic Models

**Figure 1:**
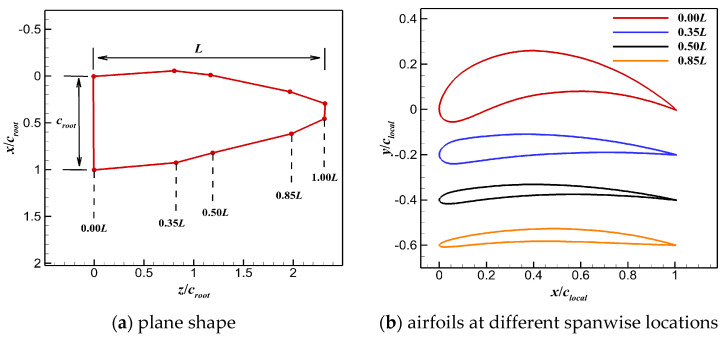
Plane shape and sectional profile of a pigeon-inspired flapping wing.

**Figure 2:**
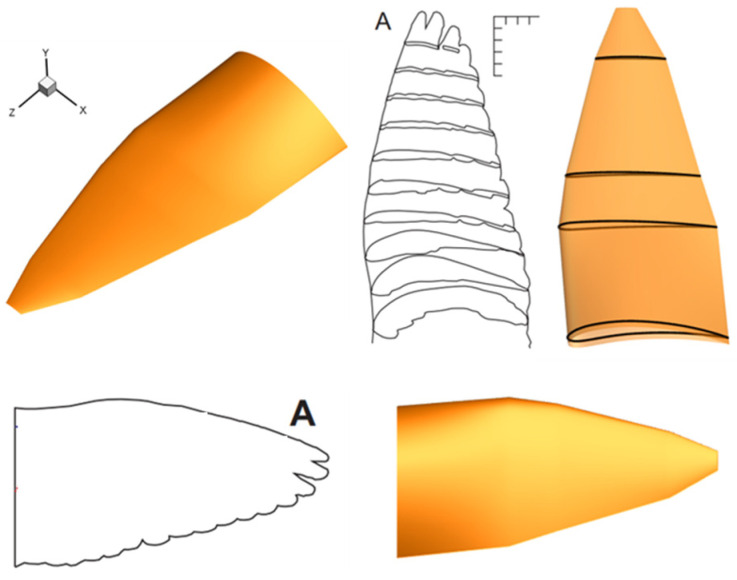
Comparison of the 3D wing model and a real pigeon wing [4].

The morphology and kinematics of a pigeon-inspired flapping wing model were reconstructed based on the detailed measurement data of a pigeon (*Columba livia domestica*) [4]. The planform of the wing model is shown in Figure 1a. The chord distribution over the span and the aspect ratio were simplified according to the reference data. The airfoils (NACA 4-digit modified series) used for generating the 3D wing model are shown in Figure 1b. The 3D wing model and profile airfoils at different spanwise locations are shown in Figure 2.

**Figure 3:**
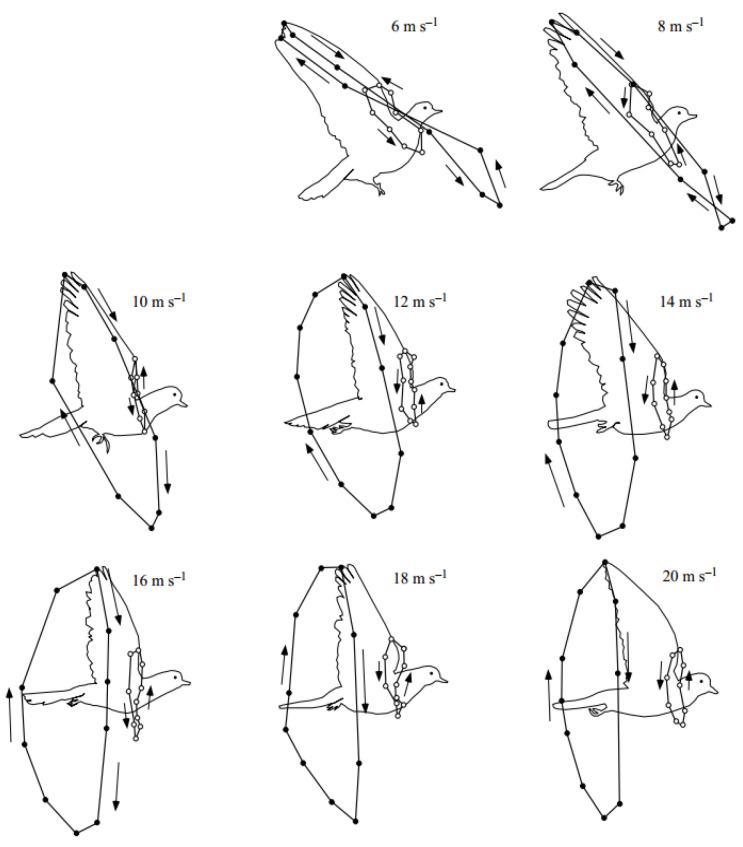
Movement trajectories of the wingtip and wrist of a pigeon at different flight speeds [25].

The 3D wingbeat kinematics of a flying pigeon was extracted using a high-speed video taken in a wind tunnel under speeds ranging from 6 m/s to 20 m/s [25]. The movement trajectories of the wingtips and wrists under different wind speeds are shown in Figure 3. This paper focuses on the effects of different movements on the aerodynamic performance of pigeons during cruising. Thus, the movement of the wingtip and wrist under the flight speed of 12 m/s, which is close to the cruising speed, was chosen to reconstruct the kinematics of pigeon wings. Correspondingly, the angle between the horizontal plane and the pigeon’s body was 13°, the flapping frequency of the wings was 6.5 Hz, and the downstroke ratio during a flapping cycle was ca. 0.53.

**Figure 4:**
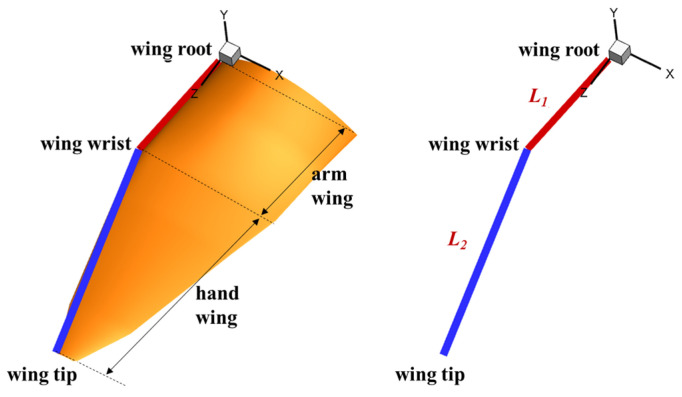
Two-joint arm model.

**Figure 5:**
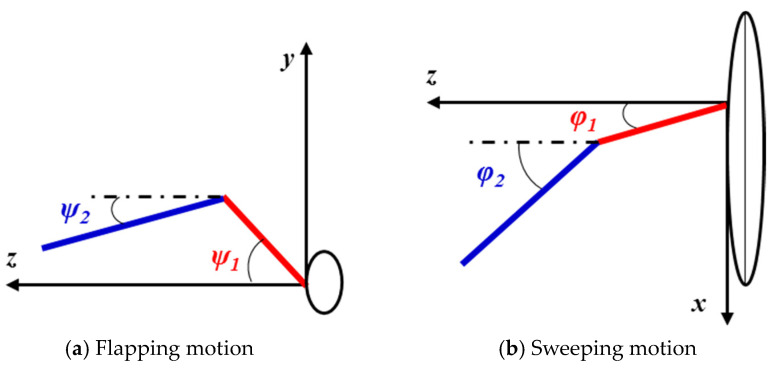
Kinematics of the two-jointed arm model defined by *Ψ*_1_, *Ψ*_2_, *φ*_1_, and *φ*_2_.

**Figure 6:**
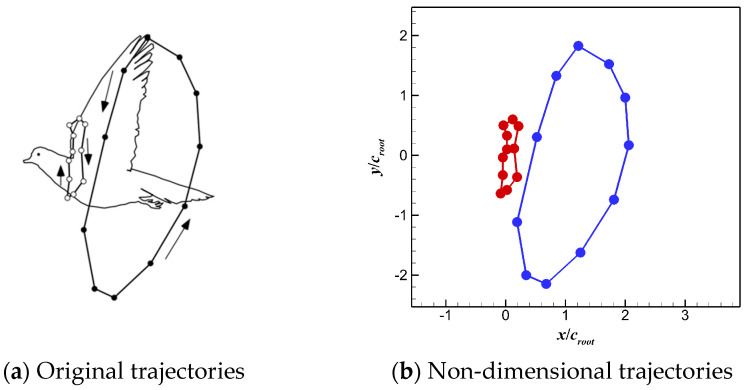
Side view of trajectories of the wingtip and the wrist of a pigeon wing.

The skeletal structure of a pigeon wing can be defined as a three-jointed arm model. It can be further simplified as a two-joint arm model [2], since the motion between the humerus and the ulna is not obvious during horizontal forward flight. As shown in Figure 4, the arm model with two rigid-joint rods is located at the leading edge of the wing, and the lengths of the arm wing and the hand wing are *L*_1_ and *L*_2_, respectively. Figure 5 shows the front view and the projected view of the two-joint arm system whose kinematics can be defined by four Euler angles (*Ψ*_1_, *Ψ*_2_, *φ*_1_, and *φ*_2_). *Ψ*_1_ and *Ψ*_2_ are used to describe the flapping motion. During the flapping cycle, both the arm wing and the hand wing undergo the forward and backward motion called the sweeping motion. In this part, *φ*_1_ and *φ*_2_ are used to describe the sweeping motion, which results in dynamic changes of the wingspan. The side view of trajectories of the wingtip and the wrist is shown in Figure 6.

**Figure 7:**
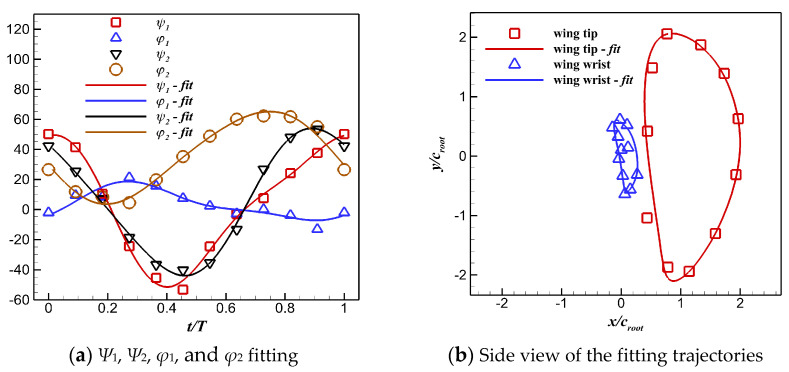
Time histories of *Ψ*_1_, *Ψ*_2_, *φ*_1_, and *φ*_2_ fitted using Fourier series.

Based on the *x*, *y* coordinates of the wrist and the wingtip and the length values of *L*_1_ and *L*_2_, the *z*-coordinates of the wrist and the wingtip can be obtained according to Equation (1), and then the discrete 3D coordinates of the wingtip (*x*_1_, *y*_1_, and *z*_1_) and the wrist (*x*_2_, *y*_2_, and *z*_2_) during a flapping cycle can be obtained. Furthermore, the values of *Ψ*_1_, *Ψ*_2_, *φ*_1_, and *φ*_2_ can be calculated according to Equation (2). Finally, the time histories of these angles are fitted via Fourier series to obtain their functions with respect to time t, as presented in Equations (3)–(7). Figure 7 shows that the fitted time histories agree with the original discrete angles; therefore, the motion of the pigeon wing can be well-reconstructed by using the simplified two-joint arm model.

(1) $$x_{1}^{2}+y_{1}^{2}+z_{1}^{2}=L_{1}^{2}\;{\left(x_{2}-x_{1}\right)}^{2}+{\left(y_{2}-y_{1}\right)}^{2}+{\left(z_{2}-z_{1}\right)}^{2}=L_{2}^{2}$$

(2) $$\begin{array}{l} \psi_{1}=\arctan(\frac{y_{1}}{z_{1}})\\ \psi_{2}=\arctan(\frac{y_{2}-y_{1}}{z_{2}-z_{1}})\\ \varphi_{1}=\arctan(\frac{x_{1}}{z_{1}})\\ \varphi_{2}=\arctan(\frac{x_{2}-x_{1}}{z_{2}-z_{1}}) \end{array}$$

(3) $$\begin{array}{ll} \psi_{1}(t) & =3.763+47.46\cos(\omega t)-7.815\sin(\omega t)-3.709\cos(2\omega t)-1.185\sin(2\omega t)\\ & +0.7896\cos(3\omega t)+0.3023\sin(3\omega t)-1.519\cos(4\omega t)-1.1\sin(4\omega t) \end{array}$$

(4) $$\begin{array}{ll} \psi_{2}(t) & =1.759+49.54\cos(\omega t)-4.569\sin(\omega t)-2.314\cos(2\omega t)-4.557\sin(2\omega t)\\ & -2.433\cos(3\omega t)+1.043\sin(3\omega t)+0.8314\cos(4\omega t)+0.5763\sin(4\omega t) \end{array}$$

(5) $$\begin{array}{ll} \varphi_{1}(t)= & 5.38-7.053\cos(\omega t)+13.56\sin(\omega t)-1.809\cos(2\omega t)+1.285\sin(2\omega t)\\ & +0.2645\cos(3\omega t)+1.13\sin(3\omega t) \end{array}$$

(6) $$\varphi_{2}(t)=34.48+1.687\cos(\omega t)-32.69\sin(\omega t)-3.151\cos(2\omega t)-3.544\sin(2\omega t)$$

### 2.2. Aerodynamic Performance

Time-averaged lift coefficient *C_L,mean_*, thrust coefficient *C_T,mean_*, and power coefficient *C_P,mean_* were adopted to evaluate the aerodynamic performance of the flapping wing. They are defined as follows:

(7) $$C_{L,mean}=\frac{\frac{1}{T}\int_{t}^{t+T}F_{y}(t)dt}{0.5\rho U_{\infty }^{2}S}=\frac{1}{T}\int_{t}^{t+T}C_{L}dt$$

(8) $$C_{T,mean}=\frac{\frac{1}{T}\int_{t}^{t+T}-F_{x}(t)dt}{0.5\rho U_{\infty }^{2}S}=\frac{1}{T}\int_{t}^{t+T}C_{T}dt$$

(9) $$C_{P,mean}=\frac{\frac{1}{T}\int_{t}^{t+T}P_{input}(t)dt}{0.5\rho U_{\infty }^{3}S}=\frac{1}{T}\int_{t}^{t+T}C_{P}dt$$

where *F_y_*(*t*) and *F_x_*(*t*) are the instantaneous forces in the *y*-direction and the *x*-direction at time *t*, respectively, and *C_L_* and *C_T_* are the corresponding non-dimensional coefficients. The instantaneous consumed power *P_input_* and its coefficient *C_P_* can be expressed as follows:

(10) $$P_{input}=\sum_{i=1}^{N}\left(F_{x,i}(t)u_{g,i}+F_{y,i}(t)v_{g,i}+F_{z,i}(t)w_{g,i}\right)$$

(11) $$C_{P}=P_{input}/\frac{1}{2}\rho U_{\infty }^{3}S$$

where *i* and *N* denote the number of the cell and the total number of cells, respectively.

Lift efficiency $\eta_{L}$ and propulsive efficiency $\eta_{T}$ can be defined as follows:

(12) $$\eta_{L}=\frac{C_{L,mean}}{C_{P,mean}}$$

(13) $$\eta_{T}=\frac{C_{T,mean}}{C_{P,mean}}$$

### 2.3. Numerical Simulation Methods

#### 2.3.1. Flow Solver

In this work, an in-house cell-centered moving multi-block CFD solver based on the finite volume method was used to solve the 3D Unsteady Reynolds-averaged Navier–Stokes (URANS) equations. The SA-DDES model was adopted to close the URANS equations. The Jameson–Schmidt–Turkel (JST) scheme was used for the spatial discretization. A dual time-stepping method was adopted for time advancing, and the lower–upper symmetric Gauss–Seidel (LU-SGS) scheme was implemented to accelerate the convergence of pseudo-time-stepping.

The NACA0012 airfoil unsteady flow simulation case with sinusoidal pitch oscillation was performed under the conditions of freestream velocity *U_∞_* = 14 m/s and chord-based Reynolds number *Re* = 1.35 × 10^5^ in order to evaluate the accuracy of our CFD solver. The kinematic parameters of the pitching motion were as follows: reduced frequency *k* = 0.1, pitching amplitude *α_m_* = 15°, and mean pitching angle *α*_0_ = 10°.

**Figure 8:**
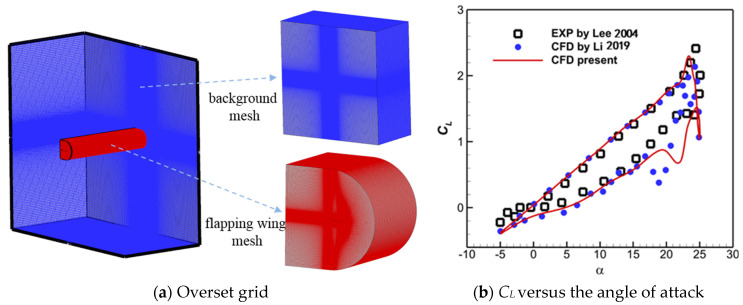
Grid and simulation results for the NACA0012 pitching case [26,27].

As depicted in Figure 8a, the computational overset grid contained a 5.72-million-cell background grid and a 9.81-million-cell flapping wing grid, with the value of *y*^+^ being less than 1.0. Figure 8b shows the hysteresis curve of the lift coefficient obtained via CFD simulations. As one can see, the overall trends of instantaneous lift coefficient *C_L_* for the present simulation were consistent with the experimental data [26] and the reference numerical results [27]. Therefore, the present solver can be used to predict the unsteady aerodynamics for a flapping wing accurately.

#### 2.3.2. Convergence Study of Grid and Time Resolution

In this section, a convergence study of grid and time resolution was performed to choose a suitable mesh and time step. The flow conditions were as follows: *U_∞_* = 12 m/s, *Re* = 9.2 × 10^4^, flapping frequency *f* = 6.5 Hz, and angle of attack AoA = 13°.

**Figure 9:**
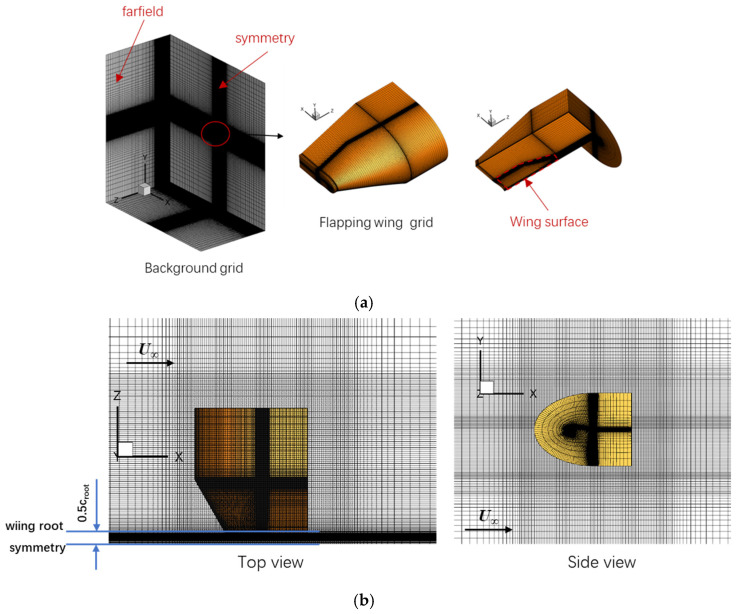
The medium grid. (**a**) Overset grid. (**b**) Different views of the flapping wing grid in the partial background grid.

**Figure 10:**
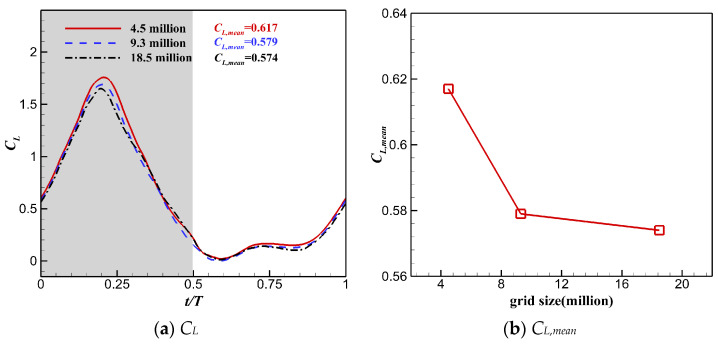
Instantaneous lift coefficient *C_L_* and time-averaged lift coefficient *C_L,mean_* computed using all the grids.

The grid convergence study was performed using three sets of grids with different densities. The coarse, medium, and fine grids had 4.2 million, 8.5 million, and 16.7 million cells, respectively. Figure 9 shows the medium grid. In this study, the wing root was 0.5 *c*_root_ from the symmetry plane, where *c*_root_ is the chord length at the wing root. Detailed parameters for the three different background grids and flapping wing grids are shown in Table 1 and Table 2. Figure 10 shows the instantaneous lift coefficient *C_L_* and the time-averaged lift coefficient *C_L,mean_* computed using all the grids. Although *C_L_* and *C_L,mean_* of the medium mesh deviated slightly from those of the fine mesh, the fine grid was selected for the following simulation to better capture the vortex structure of the flow field and reveal the flow mechanism.

**Figure 11:**
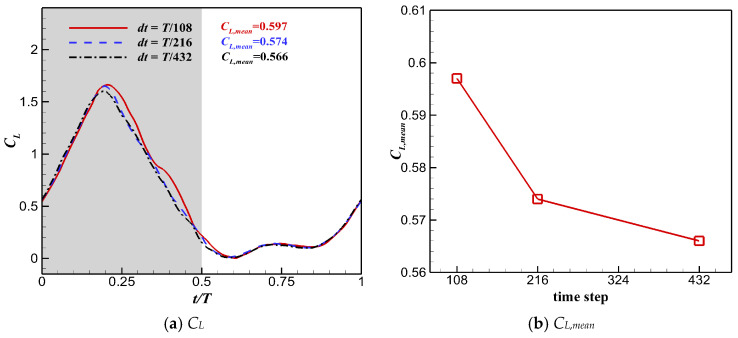
Instantaneous lift coefficient *C_L_* and time-averaged lift coefficient *C_L,mean_* computed using different time steps.

The time step sensitivity test was performed using the fine grid. Unsteady simulation was performed with a flapping cycle divided into 108, 216, and 432 time steps. Each case was calculated for 5 cycles to ensure convergence. The *C_L_* and *C_L,mean_* obtained using different time steps are shown in Figure 11. The difference in *C_L_* and *C_L,mean_* between 432 time steps and 216 time steps was much smaller than that between 216 time steps and 108 time steps, meaning good time resolution can be achieved using 432 time steps in the flapping cycle. Hence, 432 time steps during the cycle were adopted to perform the following simulations.

## 3. Results and Discussion

In this section, the effects of wing kinematics on aerodynamic performance and flow patterns of the pigeon-inspired flapping wing are numerically studied in detail.

### 3.1. Effect of the Twisting Motion on Aerodynamics

Due to the significant effects on the thrust and lift of the flapping wing, the twisting motion should be considered in the research. Nevertheless, it is difficult to measure the twisting motion of the wing for flying birds in an uncontrollable environment, thus the twisting motion was not considered in Section 2. Table 3 lists the numerical simulation results of pigeon wings at 12 m/s without considering the twisting motion. The wings had a time-averaged lift coefficient of 0.574, which can support a weight of 446 g. However, the weight of an adult pigeon (*Columba livia*) is about 350 g, which is much smaller than the calculated result. Since the error bar for the body angle at 12 m/s was around ±3 degrees, it was likely to result in a discrepancy between the calculated wing lift and the actual weight of the pigeon. In addition, the calculated time-averaged thrust coefficient was −0.1277 and could be translated to the drag of 99 g. Therefore, the calculated lift and thrust would be severely distorted once the twisting motion of the wing was ignored.

**Figure 12:**
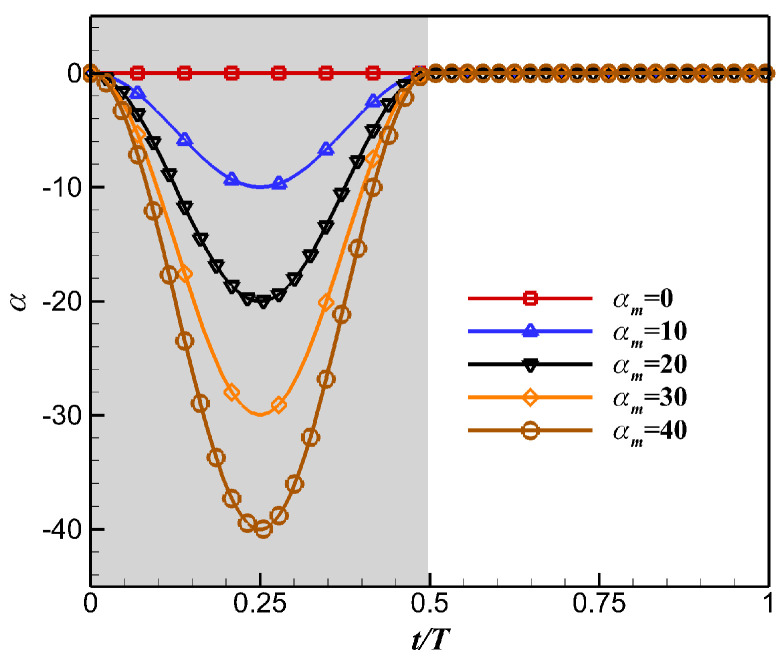
The twisting motion of the wingtip under different amplitudes *α_m_*.

In this article, a parametric cosine law of the twisting motion was applied during the wing downstroke, since the sweeping motion plays a dominant role during the upstroke. The wing’s spanwise twist distribution was linear, the root twist angle was fixed at 0°, and the wingtip twist angle was defined as Equation (14). The twisting motion of the wingtip under different amplitudes *α_m_* is shown in Figure 12.

(14) $$\begin{array}{ll} \alpha =0.5\alpha_{m}(\cos(2\omega t)-1) & t<0.5T\\ \alpha =0^{\circ } & t>0.5T \end{array}$$

**Figure 13:**
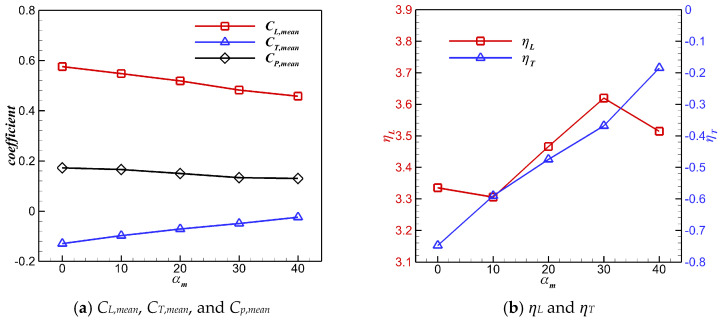
Effects of *α_m_* on *C_L,mean_*, *C_T,mean_*, *C_p,mean_*, *ƞ_L_*, and *ƞ_T_*.

The influence of different twist amplitudes *α_m_* on the aerodynamic performance of the wing with pigeon-inspired kinematics is shown in Figure 13. With the increase in *α_m_*, *C_L,mean_* and *C_P,mean_* continued to decrease, while *C_T,mean_* continued to increase. Compared with the wing without considering the twisting motion, lift efficiency *ƞ_L_* and propulsive efficiency *ƞ_T_* of the flapping wing were significantly improved when *α_m_* was greater than 10°. When *α_m_* was 40°, *C_L,mean_* and *C_T,mean_* of the flapping wing were 0.458 and −0.0241, respectively, and could be translated into the lift of 356 g and the drag of 18.6 g. The lift of the flapping wing at *α_m_* = 40° was very close to the weight of the pigeon, but the thrust was still slightly insufficient. Dynamic twisting reduced the time-averaged lift and power consumption of the flapping wing, but enhanced the time-averaged thrust significantly.

**Figure 14:**
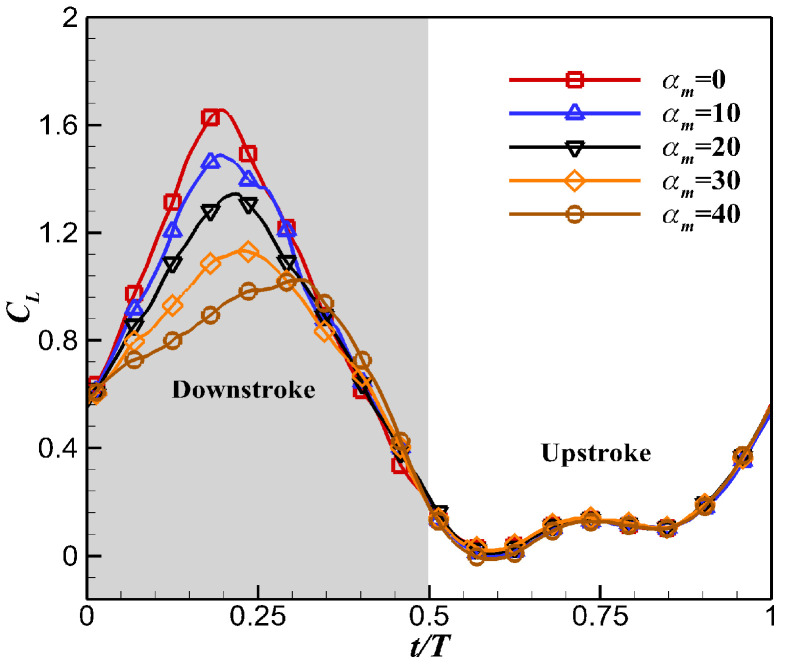
Effects of α_m_ on instantaneous lift coefficient *C_L_*.

The instantaneous lift coefficients *C_L_* under different *α_m_* during the flapping cycle are shown in Figure 14. The dynamic twisting motion mainly acted during the downstroke, while it had little effect on the aerodynamic performance during the upstroke. The effective angle of attack of the flapping wing decreased with the increase in *α_m_* during the downstroke, which resulted in a significant reduction in the instantaneous lift peak and a drop in lift performance.

**Figure 15:**
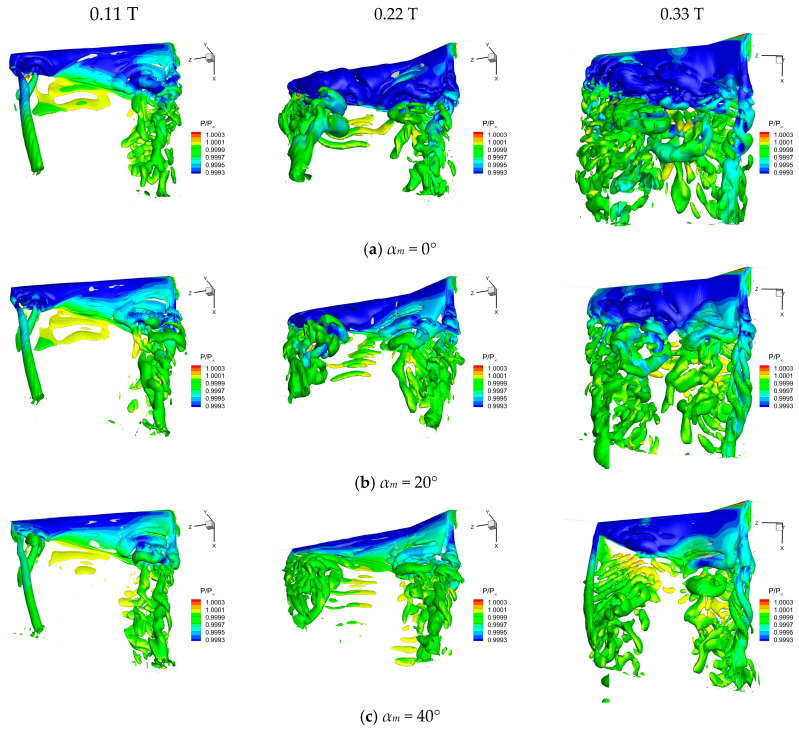
Flow field vortex structure on the upper surface of a flapping wing under different *α_m_*.

Flow field vortex structures of the flapping wing at 0.11 *T*, 0.22 *T*, and 0.33 *T* under different α_m_ are depicted in Figure 15. It is obvious that the root vortex and the wingtip vortex were induced during the downstroke under the same *α_m_*, while the leading-edge vortex (LEV) formed on the upper surface of the wing near the tip and gradually expanded toward the inner wing, eventually shedding towards the trailing edge. Further, *α_m_* has a significant effect on the evolution of the LEV on the wing’s upper surface. As *α_m_* increased, the LEV formed later and had low strength and small size. The lower pressure on the upper surface induced by the LEV was significantly weakened with the increase in *α_m_*. Besides, a significant reduction of the positive pressure on the lower surface occurred due to the decrease in *α_m_*, resulting in a decline in the lift of the flapping wing.

**Figure 16:**
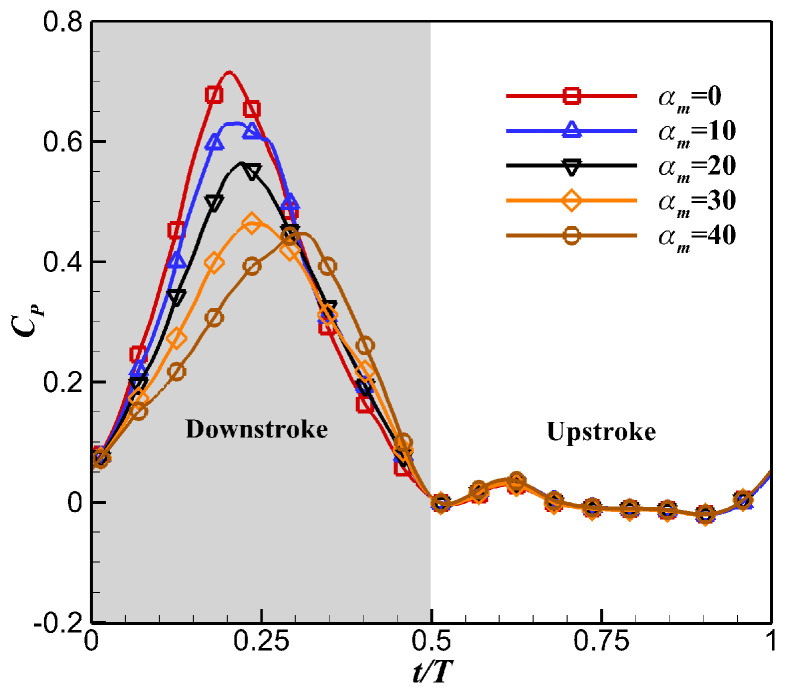
Effects of *α_m_* on instantaneous power coefficient *C_P_*.

The instantaneous power coefficients *C_P_* obtained under different *α_m_* during the flapping cycle are shown in Figure 16. With the increase in *α_m_*, the peak of the *C_P_* gradually decreased, leading to a significant reduction in the average power consumption *C_P,mean_*. Although the dynamic twisting motion reduced the time-averaged lift of the flapping wing, it promoted the lift efficiency by reducing the power consumption.

**Figure 17:**
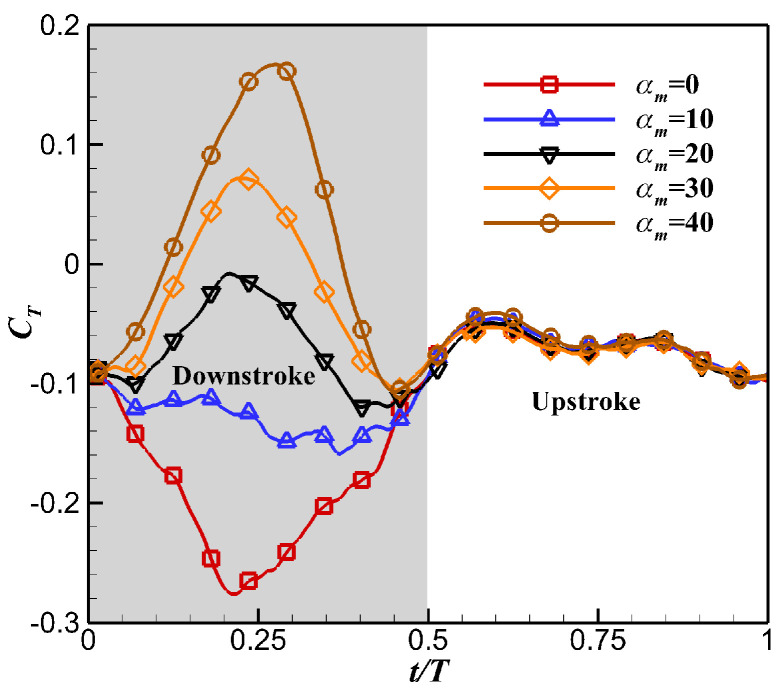
Effects of α_m_ on instantaneous thrust coefficient *C_T_*.

**Figure 18:**
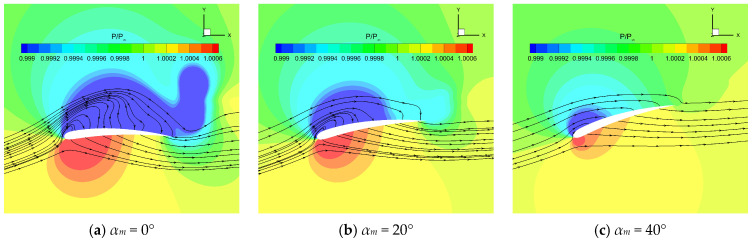
Pressure distribution at the half-span of the flapping wing under different *α_m_* when t = 2.5T.

The time history of instantaneous thrust coefficient *C_T_* under different *α_m_* is shown in Figure 17. The increase in *α_m_* significantly changed the instantaneous thrust during the downstroke. As *α_m_* increased, the flapping wing changed from producing a drag peak to generating a thrust peak in the middle of the downstroke; thus, the time-averaged thrust performance was greatly improved. Figure 18 shows the pressure distribution at the half-span of the flapping wing under different *α_m_* when t = 0.25 *T*. The increase in *α_m_* reduced the angle of attack of the flapping wing, which reduced the LEV on the upper surface. As a result, the area of the low-pressure region on the wing’s upper surface was reduced, which was not conducive to the generation of large lift. However, it should be noticed that the increase in *α_m_* could significantly change the direction of the pressure force through the deflection of the wing, which is conducive to generating a larger forward pressure force component, thereby increasing the thrust. As shown in Table 4, with the increase in *α_m_*, the time-averaged thrust of the flapping wing increased and the time-averaged power consumption decreased, which significantly improved the propulsion efficiency of the flapping wing.

### 3.2. Effect of the Sweeping Motion on Aerodynamics

In this section, the pigeon-inspired kinematics with the twisting motion and *α_m_* = 40° was adopted to further explore the aerodynamic effect of the sweeping motion on a flapping wing.

**Figure 19:**
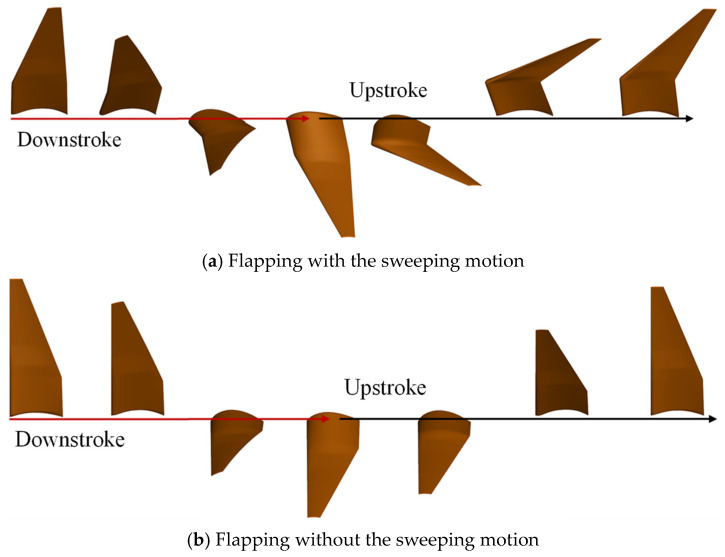
The motion of the flapping wing with or without sweeping.

During the flapping cycle, both the arm wing and the hand wing undergo the forward and backward sweeping motion, which results in dynamic changes of the wingspan. To investigate the effect of the sweeping motion on the aerodynamic performance of a flapping wing, two motion modes were constructed for a comparative study. One was to keep the sweeping motion of the hand wing and the arm wing, the wingspan of which could dynamically change during the flapping cycle as shown in Figure 19a. The other was to remove the sweeping motion, and the wingspan was kept fixed during the whole flapping cycle, as shown in Figure 19b.

Table 5 lists a comparison of the time-averaged aerodynamic parameters of the flapping wing with and without the sweeping motion. One can see that the lift performance was greatly enhanced for the flapping wing while maintaining the sweeping motion. The value of *C_L,mean_* was increased by 22.1% and the value of *C_P,mean_* was reduced by 43.2% for the wing with the sweeping motion. The lift efficiency increased from 1.64 to 3.52 (115%) when the sweeping motion was considered. Although there was a decrease in thrust, the lift performance could be greatly improved while adopting the sweeping motion.

**Figure 20:**
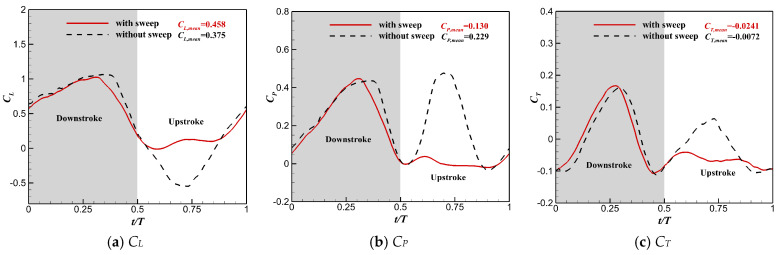
Comparison of instantaneous aerodynamic coefficients for the flapping wing with or without the sweeping motion.

Figure 20 shows the time history of *C_L_*, *C_T_*, and *C_P_* during the flapping cycle. During the downstroke, the relative sweeping motion between the hand wing and the arm wing was not obvious due to the small sweeping amplitude of the whole flapping wing. However, this relative motion was very obvious during the upstroke, since the hand wing performed a large amplitude backward sweeping motion. Therefore, the difference in the aerodynamic performance of the flapping wing obtained by considering the sweeping motion and without considering it was mainly reflected during the upstroke. When the sweeping motion was considered, there was an obvious spanwise contraction during the upstroke for the flapping wing, resulting in a significant decrease in the instantaneous negative lift peak. The improvement of the instantaneous lift performance significantly reduced the instantaneous power consumption of the flapping wing during the upstroke. In addition, the peak thrust of the flapping wing reduced during the upstroke when considering the sweeping motion, which had a negative effect on the thrust.

**Figure 21:**
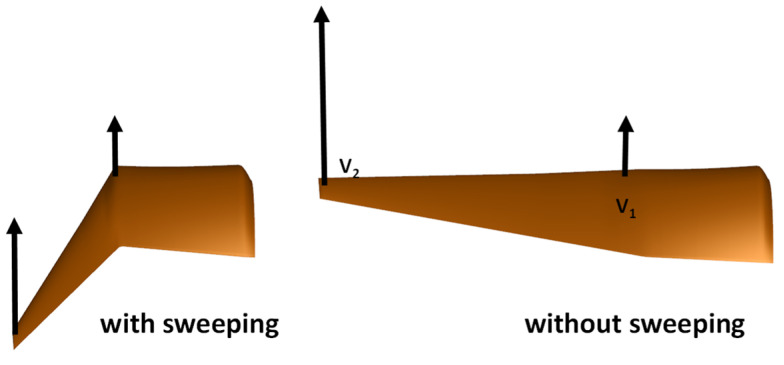
Comparison of the upward flapping speed at different spanwise locations of the flapping wing when t = 0.75 T.

**Figure 22:**
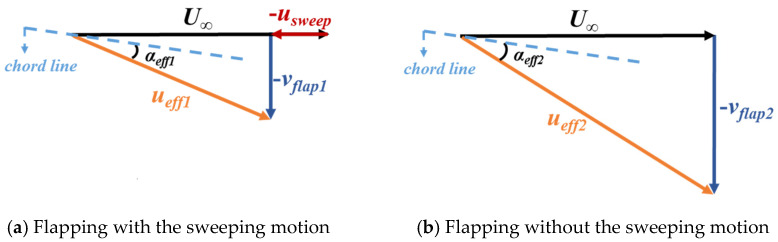
Effective incoming velocity *u_eff_* and negative effective angle of attack *α_eff_* at the tip of the flapping wing when t = 0.75 T.

As shown in Figure 21, the dynamic sweeping motion reduced the wingspan, resulting in a lower upward movement velocity from the wrist to the wingtip than that of the flapping wing without the sweeping motion during the upstroke. Moreover, the hand wing had a larger backward movement during the upstroke for the flapping wing with the sweeping motion. As a result, the horizontal velocity of the incoming flow relative to the flapping wing decreased, leading to the decrease in the effective incoming velocity of the flapping wing in the same spanwise locations at the arm wing. Figure 22 shows the motion analysis of the wingtip for flapping wings with and without the sweeping motion in the middle of the upstroke. Compared with the flapping wing without the sweeping motion, the wing with the sweeping motion had a lower effective incoming velocity *u_eff_* and a negative effective angle of attack *α_eff_* at the wingtip. This pattern was also suitable for other locations from the wrist to the wingtip.

**Figure 23:**
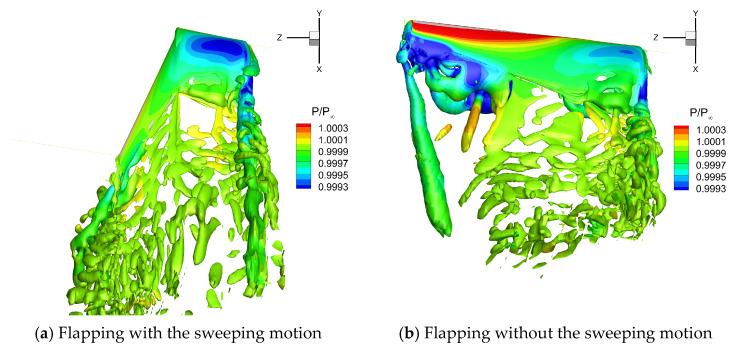
Flow field vortex structure on the upper surface of the flapping wing when t = 0.75 T.

**Figure 24:**
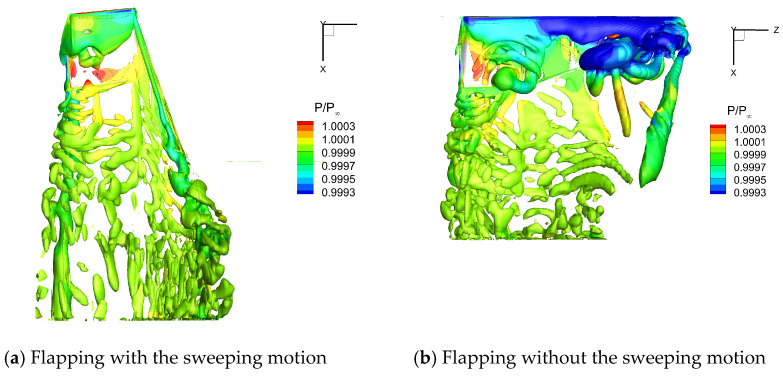
Flow field vortex structure on the lower surface of the flapping wing when t = 0.75 T.

Figure 23 and Figure 24 show the flow field vortex structure on the upper and lower surfaces of the flapping wing in the middle of the upstroke (t = 0.75 T), respectively. For the flapping wing without the sweeping motion, larger *α_eff_* and *u_eff_* led to a strong positive pressure on the upper surface. In addition, a stronger LEV structure was generated on the lower surface, leading to a large low-pressure region on the lower surface. The pressure difference between the upper and lower surfaces of the flapping wing was relatively large, which led to a large negative lift for the flapping wing without sweeping. However, *α_eff_* and *u_eff_* of the flapping wing with the sweeping motion were small, which significantly weakened the positive pressure on the upper surface, which was harmful to lift. Meanwhile, the LEV structure of the lower surface was eliminated significantly, and the low pressure on the lower surface was reduced, thus reducing the negative lift of the flapping wing. In addition, the sweeping motion reduced the area of the flapping wing. As a result, the wing area was only 60% of that without the sweeping motion when t = 0.75 T, which was also important for the decrease in negative lift during the upstroke.

In summary, the sweeping motion can effectively improve the lift performance of a flapping wing. The lifting efficiency of a flapping wing is significantly improved by reducing the negative lift peak and reducing the power consumption. The disadvantage is that the sweeping motion would degrade the propulsive performance of the flapping wing during the upstroke.

## 4. Conclusions

In this article, the effects of wing kinematics on the aerodynamic performance for a pigeon-inspired flapping wing were investigated through a hybrid RANS/LES method. The dynamic geometric shape of a flapping wing was reconstructed based on data of the pigeon wing profile. The 3D wingbeat kinematics of a flying pigeon was extracted from the motion trajectories of the wingtip and the wrist during cruise flight. The effects of wing twisting and the sweeping motion on the aerodynamic performance and flow pattern were analyzed in detail. The main conclusions are as follows:

(1) The dynamic twisting motion leads to a decrease in lift, an increase in thrust, and a decrease in power consumption for the flapping wing during the downstroke. The twisting motion can weaken the LEV on the upper surface of the wing during the downstroke by reducing the effective angle of attack, thereby significantly reducing the time-averaged lift and power consumption. In addition, dynamic twisting can deflect the wing profile, which causes the pressure difference between the upper and lower surfaces to generate a forward thrust component, leading to an increase in transient thrust for the flapping wing.

(2) The sweeping motion reduces the wing area and weakens the LEV on the lower surface of the wing, which significantly increases the lift and reduces the aerodynamic power consumption during the upstroke, leading to high lift efficiency. On the one hand, the sweeping motion reduces the negative effective angle of attack and the effective incoming velocity of the flapping wing, which can not only weaken the positive pressure on the upper surface that is harmful to the lift, but also basically eliminate the LEV structure on the lower wing surface and reduce the negative pressure on the lower surface, thus playing a role in reducing the negative lift during the upstroke. On the other hand, the sweeping motion reduces the wingspan of the flapping wing, resulting in a reduction of the wing area. The reduction of the wing area during the upstroke also plays a role in reducing the instantaneous negative lift. Due to the reduction of the negative lift, the power consumed to generate lift for a flapping wing with the sweeping motion during the upstroke is reduced, leading to lift efficiency improvement.

In this study, the pigeon body was ignored to simplify geometry modelling, which resulted in unphysical vortices at the wing root. In addition, the existing literature data are not sufficient to construct the real twisting motion of a pigeon, and the parametric twisting motion established in this study is not sufficient to fully reveal the aerodynamic mechanism of the twisting motion of the pigeon wing. Thus, further study of these two aspects is still recommended in the future.

## Figures and Tables

**Table 1:** Parameters for the different background grids.

Parameters	Far Field Length (*c*)	Grid Cell Number	Total Grid Volume
Coarse grid	30	123 × 133 × 133	2.2 million
Medium grid	30	145 × 155 × 205	4.6 million
Fine grid	30	189 × 201 × 221	8.4 million

**Table 2:** Parameters for the different flapping wing grids.

Parameters	Grid Cell Number	Total Grid Volume	First Grid Layer Spacing (*c*)	Spacing Increase Ratio
Coarse grid	265 × 57 × 133	2.0 million	3.5 × 10^−4^	1.1
Medium grid	321 × 81 × 151	3.9 million	2.6 × 10^−4^	1.09
Fine grid	401 × 107 × 193	8.3 million	1.8 × 10^−4^	1.08

**Table 3:** Details of the baseline CFD grids.

Parameter	*C_L,mean_*	*C_T,mean_*	*C_P,mean_*
Far field radius (*c*)	0.574	−0.1277	0.173

**Table 4:** Comparison of time-averaged aerodynamic coefficients for the pigeon-inspired flapping wing with or without twisting.

Parameters	*C_L,mean_*	*C_T,mean_*	*C_P,mean_*	*ƞ* *_L_*	*ƞ* *_T_*
Without twisting	0.576	−0.1291	0.173	3.33	−0.746
With twisting	0.458	−0.0241	0.130	3.52	−0.185

**Table 5:** Comparison of the time-averaged aerodynamic performance of the flapping wing with and without the sweeping motion.

Parameters	*C_L,mean_*	*C_T,mean_*	*C_P,mean_*	*ƞ* *_L_*	*ƞ* *_T_*
Without the sweeping motion	0.375	−0.0072	0.229	1.64	−0.031
With the sweeping motion	0.458	−0.0241	0.130	3.52	−0.185

## Data Availability

The original contributions presented in this study are included in the article. Further inquiries can be directed to the corresponding author.
